# High-resolution isotopic evidence for a potential Saharan provenance of Greenland glacial dust

**DOI:** 10.1038/s41598-018-33859-0

**Published:** 2018-10-22

**Authors:** Changhee Han, Soon Do Hur, Yeongcheol Han, Khanghyun Lee, Sungmin Hong, Tobias Erhardt, Hubertus Fischer, Anders M. Svensson, Jørgen Peder Steffensen, Paul Vallelonga

**Affiliations:** 1 Department of Ocean Sciences, Inha University, 100 Inha-ro, Michuhol-gu, Incheon 22212 Korea; 2 Korea Polar Research Institute, 26 Songdomirae-ro, Yeonsu-gu, Incheon 21990 Korea; 3 Climate and Environmental Physics, Physics Institute & Oeschger Center for Climate Change Research, University of Bern, Sidlerstrasse 5, 3012 Bern, Switzerland; 4 Center for Ice and Climate, Niels Bohr Institute, University of Copenhagen, Julian Maries Vej 30, 2100 Copenhagen, Denmark

## Abstract

Dust concentrations in Greenland ice show pronounced glacial/interglacial variations with almost two orders of magnitude increase during the Last Glacial Maximum. Greenland glacial dust was previously sourced to two East Asian deserts: the Taklimakan and Gobi deserts. Here we report the first high-resolution Pb and Sr isotopic evidence for a significant Saharan dust influence in Greenland during the last glacial period, back to ~31 kyr ago, from the Greenland NEEM ice core. We find that during Greenland Stadials 3–5.1 (~31 to 23 kyr ago), the primary dust provenance was East Asia, as previously proposed. Subsequently, the Saharan isotopic signals emerge during Greenland Stadials 2.1a–2.1c (~22.6 to 14.7 kyr ago) and from the late Bølling-Allerød to the Younger Dryas periods (~13.6 to 12 kyr ago), coincident with increased aridity in the Sahara and efficient northward transport of dust during these cold periods. A mixing isotopic model proposes the Sahara as an important source, accounting for contribution to Greenland glacial dust of up to 50%, particularly during Greenland Stadial 2.1b and the late Bølling-Allerød to the Younger Dryas periods. Our findings provide new insights into climate-related dust provenance changes and essential paleoclimatic constraints on dust-climate feedbacks in northern high latitudes.

## Introduction

Atmospheric mineral dust (hereafter referred to as dust) is an important component of the Earth’s climate system, potentially affecting the radiative balance through reflection and absorption of both the incoming solar radiation and the outgoing infrared radiation^1,2^. Climate models have attempted to assess the radiative effects of dust on climate under present and past climatic conditions with the final goal of future prediction, using *in-situ* and remote observation dust data for the modern era and paleo-dust records in sediments and ice cores for the past^3–5^.

Recently, a stronger variability of Arctic near-surface air temperatures, an effect known as ‘Arctic amplification’, emerged as a key issue in future global climate simulations^6,7^. The main contributors to Arctic amplification are still under debate for the recent amplified warming^6,7^. As for the past Arctic amplification, recent model simulation proposed that the surface cooling effect of dust could be of similar strength as the radiative forcing due to the reduced CO_2_ during the Last Glacial Maximum (LGM; 23–19 kyr ago)^5^. However, radiative forcing varies with the spatial and vertical dust concentrations^4,5^, which depend on transport patterns from the continental dust source areas to the Arctic by atmospheric circulation^8,9^.

The LGM is well characterized by its very dust-laden atmosphere, as reflected by an approximate 20-fold increase in Greenland dust deposition compared to the Holocene^10^ potentially caused by the expansion of source areas, enhanced dust mobilization by strong winds, and increased atmospheric residence time^11,12^. However, the discrepancy between the observed and modeled dust fluxes in Greenland for the LGM still exists, possibly due to the source area changes in potential contributors to Greenland dust^4,13–15^. This highlights the importance of better understanding dust sources under different climatic conditions for reducing the uncertainty in estimates of the dust distribution in the Arctic using dust models.

Early studies confined the main dust source in the GRIP and GISP2 ice cores, drilled at the Summit in Greenland, during the cold climatic periods using mineralogical and isotopic characteristics^16–18^. They pointed to East Asian deserts (the Taklimakan and the Gobi) as the main source of the Greenland dust for all time periods in the past. However, the dominant dust source affecting glacial dust flux in Greenland still remains ambiguous. The Sahara^18,19^, a central European source^20^, or Siberian/Alaskan sources^15^ have been proposed as additional potential contributors to Greenland dust. The uncertainty in discriminating Greenland dust provenance is also large for the current climatic condition. Isotopic studies of dust in Greenland snow and ice assigned East Asia and/or the Sahara as the dominant sources^21–23^.

## Temporal variability of concentrations and isotopes of Pb and Sr

**Figure 1:**
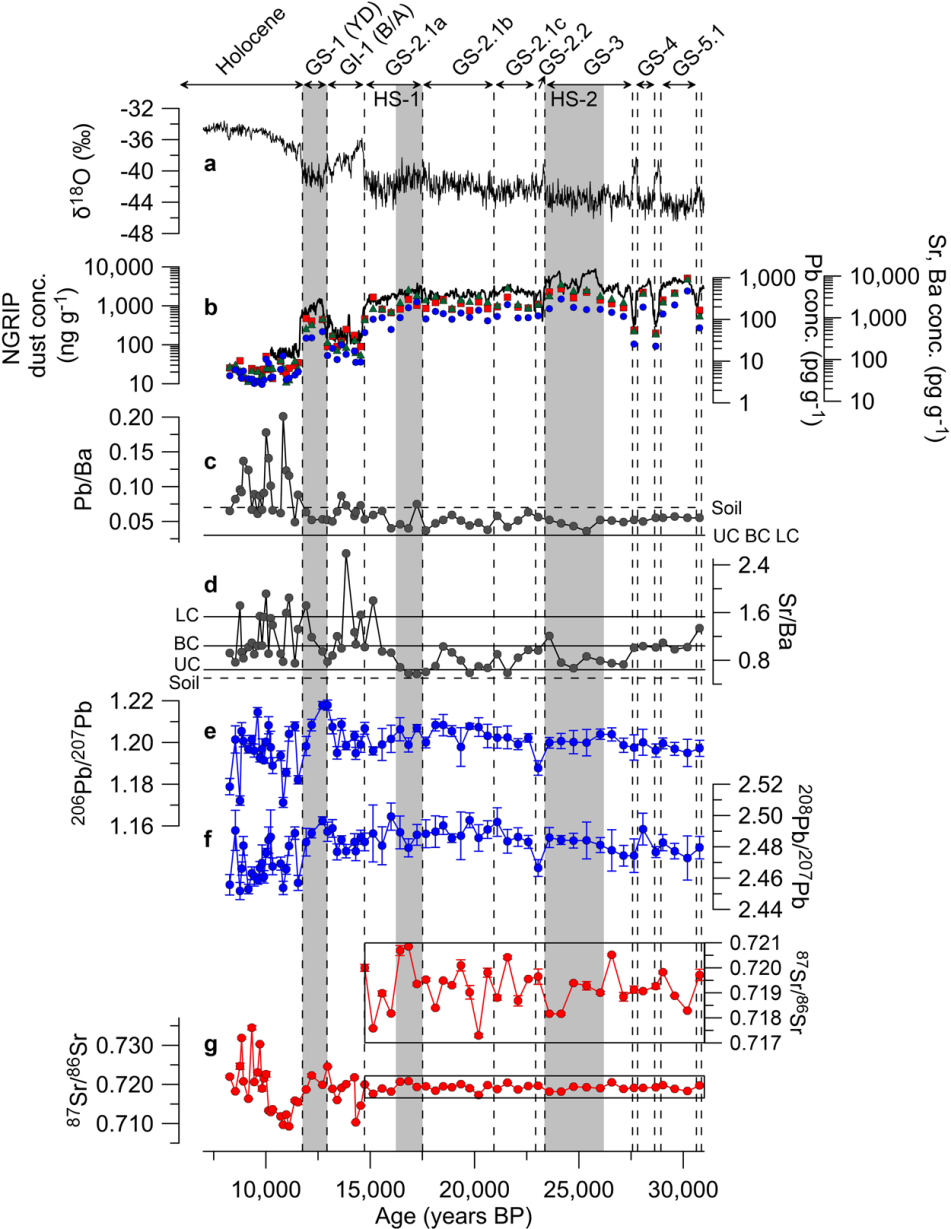
Changes in Pb, Sr and Ba concentrations, and Pb and Sr isotopic compositions from the NEEM ice core over the past ~31 kyr. (**a**) NEEM δ^18^O (Greenland temperature proxy) isotopic profile^24^ and Greenland climatic events^25^ (shown by the vertical dashed lines and shaded grey; see text) on the age scale. (**b**) Pb (blue filled circles), Sr (red filled squares) and Ba (green filled triangles) concentrations in ice of the samples (shown in a logarithmic scale). Also shown is the Greenland dust (insoluble particles) concentration record from the NGRIP ice core^34^, drilled in central Greenland (shown in a logarithmic scale). (**c**,**d**) Pb/Ba and Sr/Ba ratios in each sample. Solid and dotted lines represent the mean Pb/Ba and Sr/Ba ratios in the upper (UC), lower (LC) and bulk (BC) continental crust^28^ and soils^29^, respectively. (**e**,**f**) The observed record of ^206^Pb/^207^Pb and ^208^Pb/^207^Pb ratios. (**g**) The observed record of ^87^Sr/^86^Sr ratios in aluminosilicate fraction (see Methods and Supplementary Fig. S4).

Here we report the first high-resolution Pb and Sr isotopic records evidencing Saharan dust inputs to Greenland during certain periods of the late last glacial age (LLGA). The isotopic data were obtained in 67 sections from the 2,540-m-long North Greenland Eemian Ice Drilling (NEEM) ice core (77.45° N, 51.06° W, altitude 2,450 m above sea level (asl))^24^ with ages ranging from ~8,260 to ~30,800 years before present (referred to as BP, where ‘present’ is defined as 1950) (see Supplementary Table S1 and Methods). This time interval covers dramatic millennial climate changes (Fig. 1): the early Holocene (EH), the Younger Dryas cooling event (YD) corresponding to Greenland Stadial-1 (GS-1), the Bølling-Allerød (B/A) warming event, Greenland Interstadial-1 (GI-1), and GS-2 to GS-5.1, including the LGM of the LLGA (Fig. 1) (GS and GI numbering is according to ref.^25^). In contrast to earlier isotopic tracer studies, particularly those using Nd isotopes for which large samples (>0.5 kg) were required for high precision analysis in Greenland ice^16,17^, our Pb and Sr isotopes were determined using a sample weight of ~10 g, allowing for a much higher time resolution in isotopic records related to Greenland dust provenance changes in response to climatic conditions^26^ (see Methods).

The Pb and Sr concentrations show a high covariance between the changes in the concentrations and the climatic conditions (δ^18^O, proxy of Greenland temperature) with the lowest levels (averages of 5.2 and 62 pg g^–1^, respectively) during the EH and much higher levels (averages of 155 and 2,618 pg g^–1^) during the cold stages (GS-2 to GS-5.1) (Fig. 1a,b). Concentrations vary in association with Ba (a conservative crustal reference element) and the dust profile (Fig. 1a,b), as previously found for the dust-derived trace elements in Greenland ice^18,27^. Pb/Ba and Sr/Ba ratios are within the elemental compositions of the upper/lower continental crusts^28^ or soils^29^ during the LLGA (Fig. 1c,d), indicating that most fractions of Pb and Sr in ice originated from mineral dust. These ratios also vary significantly, notably for Pb, with some enhanced values above the crustal ratios during the B/A and EH. These variations may be the consequence of changes in the crustal mineralogy due to changes in the geographical locations of dust sources^27^ and/or non-crustal contribution, particularly from volcanoes^18^.

The isotopic compositions of Pb and Sr exhibit a significant variability over the time period back to ~31 kyr BP, with the ratios distributed in a relatively narrow range during cold climatic conditions (Fig. 1e–g). During the LLGA, the Pb isotopic composition shows a substantial level of variability (ranges, 1.1878–1.2084 for ^206^Pb/^207^Pb and 2.4664–2.4993 for ^208^Pb/^207^Pb) with more radiogenic values during GS-2 compared to GS-4 to GS-5.1, while the ^87^Sr/^86^Sr ratios vary within smaller range of values between 0.7173 and 0.7209 (see inset of Fig. 1g). The Pb and Sr isotopic variations during the LLGA are believed to be related to changes in dust sources, because the Pb/Ba and Sr/Ba ratios are within the ratios of the continental crust or soils. The Pb and Sr isotopes vary with changing climatic conditions during the B/A and YD events. They are highly variable during the EH, with ranges of 1.1713–1.2145, 2.4517–2.4905, and 0.7093–0.7345 for ^206^Pb/^207^Pb, ^208^Pb/^207^Pb, and ^87^Sr/^86^Sr, respectively. A large variability in the isotopic composition and elemental ratios of Pb, Sr and Ba during this period has been attributed to changes in dust sources and/or possible volcanic inputs^18,27^. However, the variability could be partly due to the relatively shorter time span (less than 1 year) integrated by each sample (see Supplementary Table S1), inducing seasonal differences in the strength of multiple sources between the high-dust (spring) and the low-dust (autumn/winter) concentration seasons^21,22^.

### Transition from East Asian to Saharan isotopic source signatures

**Figure 2:**
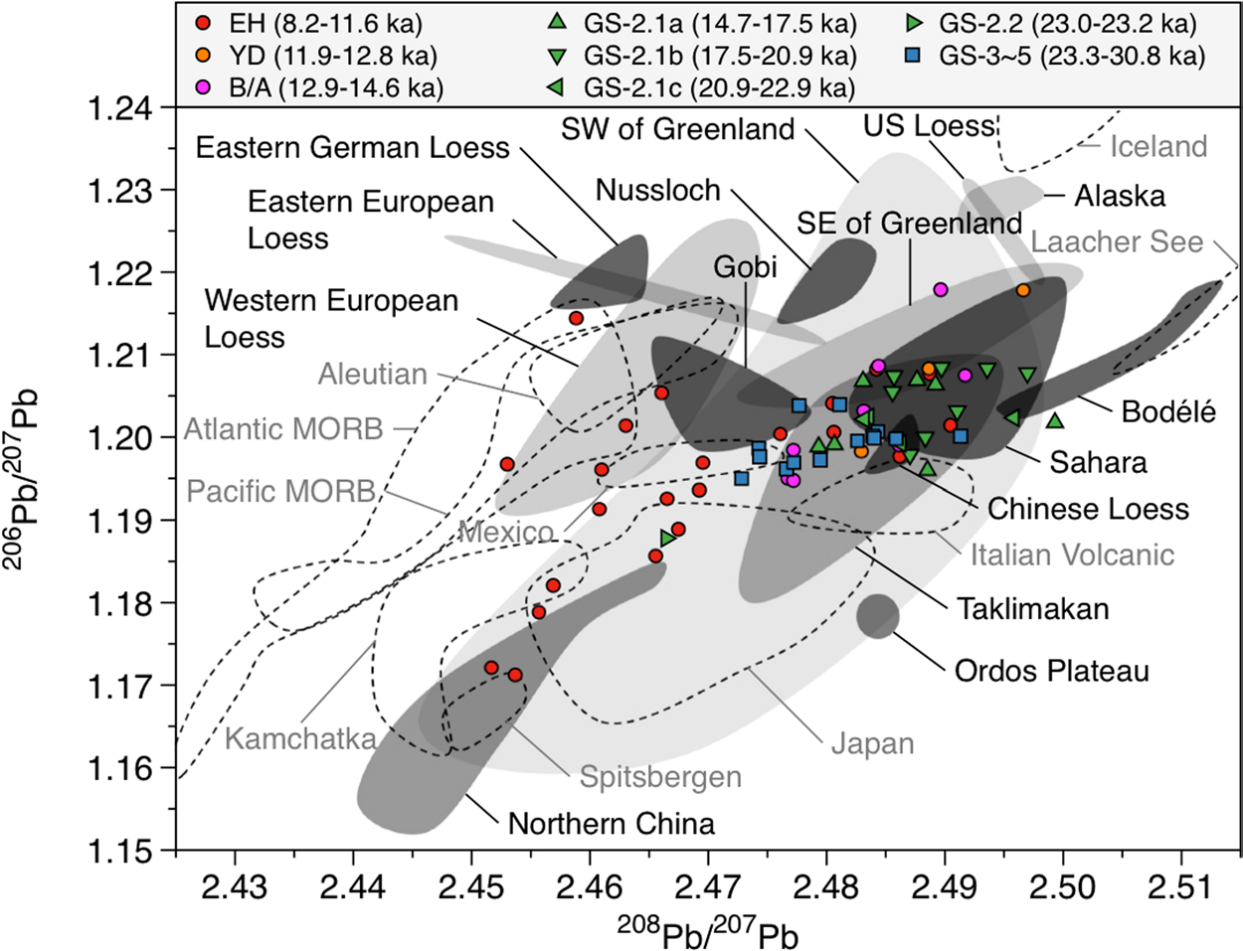
Lead isotopic compositions in the NEEM ice core. The shading and dashed areas represent the literature derived PSAs isotopic compositions defined for the potential desert sources and volcanic sources, respectively (see Supplementary Fig. S1 for details).

Pb isotopic compositions (^206^Pb/^207^Pb versus ^208^Pb/^207^Pb) in the samples are distributed along a mixing area between different potential source areas (PSAs) of dust (Supplementary Fig. S1), generally being more radiogenic during cold periods than during the EH (Fig. 2). In detail, most of the samples during GS-3 to GS-5.1, including GI-3 and GI-4, are within the range between the Taklimakan and the Gobi deserts, confirming previous findings that East Asian deserts are the main source of Greenland glacial dust^16–18^ (see Supplementary Fig. S3e). Interestingly, a different situation emerges for the Pb isotopic composition during GS-2.1a to GS-2.1c. The data tend to distribute on the edge of the Taklimakan field or within the Saharan field, having more radiogenic values (Supplementary Fig. S3c). A shift is observed in the ratios corresponding to GS-2.1b, displaying more thorogenic ratios (^208^Pb/^207^Pb: 2.4856–2.4970) (Supplementary Fig. S3c). Compared to the data during GS-3 to GS-5.1, the higher ^206^Pb/^207^Pb and ^208^Pb/^207^Pb ratios observed in GS-2.1a to GS-2.1c suggest that the Sahara could be a potential significant dust source in Greenland during these climatic periods. Subsequently, the Pb isotopic ratios in the ice are lower and move towards the Taklimakan signature during the warmer period of the B/A event, but during the late B/A and YD periods they rise again to very radiogenic values, typical for Saharan dust (Fig. 2 and Supplementary Fig. S3a).

**Figure 3:**
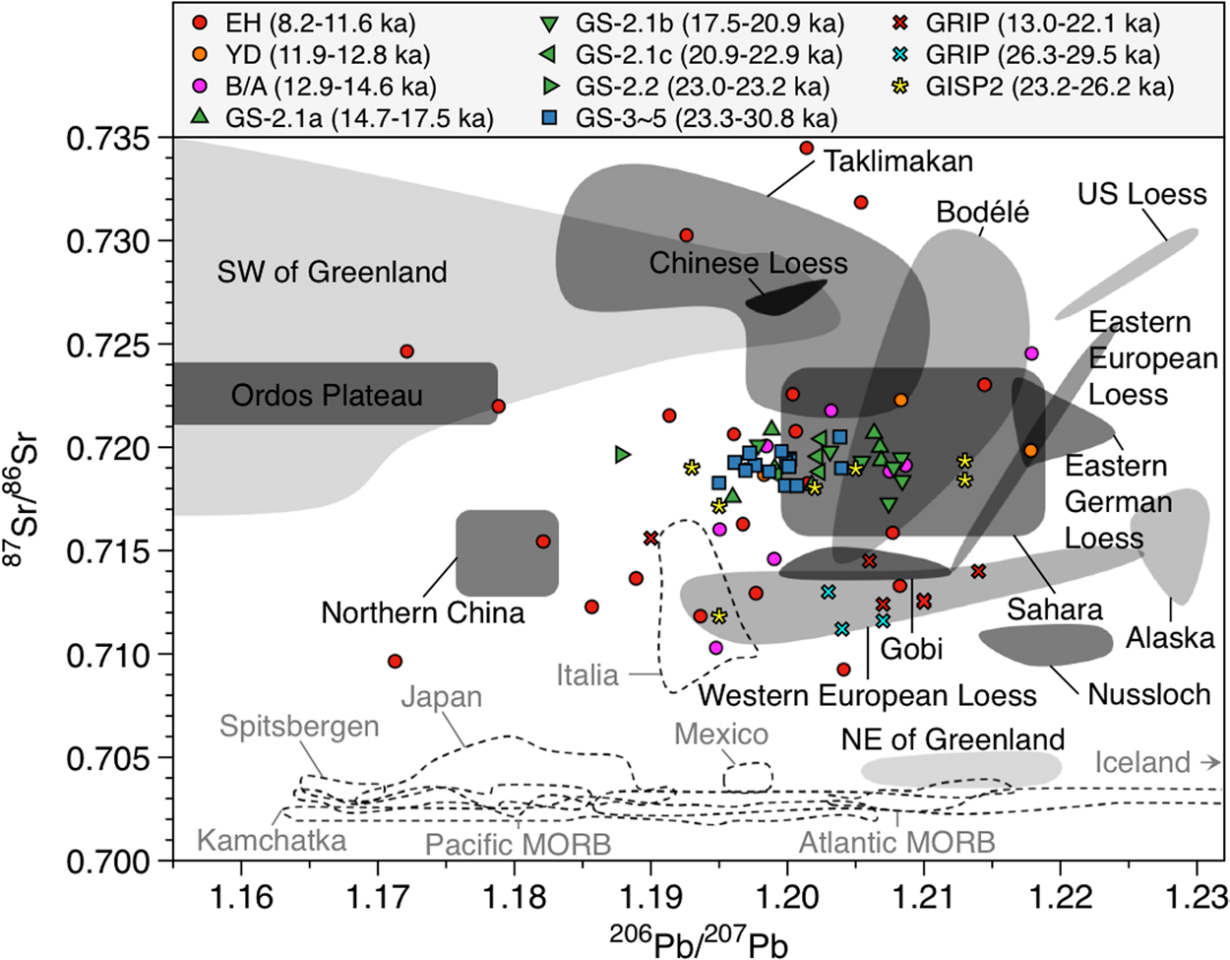
A plot of the ^87^Sr/^86^Sr vs ^206^Pb/^207^Pb in the NEEM ice core. Also included are data previously reported from the GISP2^16^ and GRIP^18^ ice cores. The shading and dashed areas represent the literature derived PSAs isotopic compositions defined for the potential desert sources and volcanic sources, respectively (see Supplementary Fig. S2 for details).

The plot of ^87^Sr/^86^Sr versus ^206^Pb/^207^Pb ratios in the samples provides additional constraints on the potential inputs of Saharan dust to Greenland (Fig. 3). The older samples (GS-3 to GS-5.1) lie on a mixture of sources from the Taklimakan, the Gobi, and northern China, except for the two samples shifting towards more radiogenic ^206^Pb/^207^Pb values in the Saharan field (sample nos. 58 (~26 kyr BP) and 59 (~26.6 kyr BP) in Supplementary Fig. S3f). The Pb isotopes for these two samples, however, plot within the Taklimakan and the Gobi Pb isotopic areas (Supplementary Fig. S3e), excluding the possibility of the Saharan dust contribution during GS-3. Comparatively, previous GISP2 data^16^ from 23.2 to 26.2 kyr BP reflect a mixing between Asian deserts and the Sahara (Fig. 3). However, the Sr isotopic composition of the GRIP samples exhibits much lower values (Fig. 3), due to no pretreatment of the samples to remove carbonate fraction in these samples (lowering ^87^Sr/^86^Sr ratios)^18^. During GS-2.1a to 2.1c, the samples are divided into three groups. First, the samples with lower radiogenic Pb isotopic ratios (the Taklimakan-dominant) are placed on a mixed field of the provenance of Asian dusts, notably between the Taklimakan and the Gobi (group A in Supplementary Fig. S3c,d). Second, the samples on the lower edge of the Saharan Pb isotopic area (mixing with the Taklimakan and the Sahara) have compositions that plot directly on a mixing area among the Taklimakan, the Gobi, and the Sahara (group B in Supplementary Fig. S3c,d), suggesting an increase in the Saharan contribution. Finally, the samples showing more radiogenic Pb isotope values (Sahara-dominated), particularly during GS-2.1b, display a systematically more Sahara-like ^87^Sr/^86^Sr versus ^206^Pb/^207^Pb signature compared to the two different groups (group C in Supplementary Fig. S3c,d). This is thought to be a consequence of the additional increase in the Saharan contribution. Interestingly, the distinct isotopic signatures for Saharan dust also emerge in the ^87^Sr/^86^Sr versus ^206^Pb/^207^Pb plot for the samples corresponding to the late B/A and YD periods (Supplementary Fig. S3b), providing insights into climatic conditions favoring dust transport out of the Sahara to Greenland. European loess deposits were previously proposed as potential candidates for Greenland glacial dust^20,30^. However, significant differences in the Pb-Sr isotopic compositions between European loess deposits and the other PSAs during cold periods rule out this hypothesis (Figs 2, 3 and Supplementary Fig. S3a–f).

The “grain-size effect” on isotope fractionation hints at further support for Saharan dust in Greenland glacial ice: the ^87^Sr/^86^Sr ratios in Chinese deserts are very high (more than 0.7260) in the fine grain-size (<2 μm) aluminosilicates due to the enrichment of clay minerals with a high content of radiogenic strontium^31^. Previous studies for Greenland glacial dust provenance compared the Sr isotopic signatures in a <5 μm size fraction of PSAs^16,17^. However, the mass mean grain size of dust in Greenland ice is within 2 μm in diameter^32^. Assuming that the Taklimakan desert is a major source area for Greenland dust as previously reported^16,17^, the Sr isotopic composition in our samples should be more radiogenic than the values we found. Thus, we argue that coupling the Sr isotopic composition in aluminosilicates with the systematic Pb isotope ratios supports a potential contribution of the Sahara to Greenland dust input during GS-2.1a to GS-2.1c and the late B/A to YD periods.

Compared to the isotopic compositions during cold periods, the Sr and Pb isotopic ratios in general shifted towards lower radiogenic values (Sahara-depleted) during the EH (Fig. 3). The isotopic compositions, mostly plotted within a mixed field between the Taklimakan, the Gobi, and northern China, imply that these PSAs are the most likely dust sources. A few isotopic excursions raise the possibility of sporadic dust inputs from proglacial floodplain in Greenland (sample nos 1 and 3 in Supplementary Fig. S3b) and a volcanic contribution with lower radiogenic ^87^Sr/^86^Sr isotopic ratios (sample nos 17 to 20 in Supplementary Fig. S3b).

### A model estimate of the Saharan contribution

**Figure 4:**
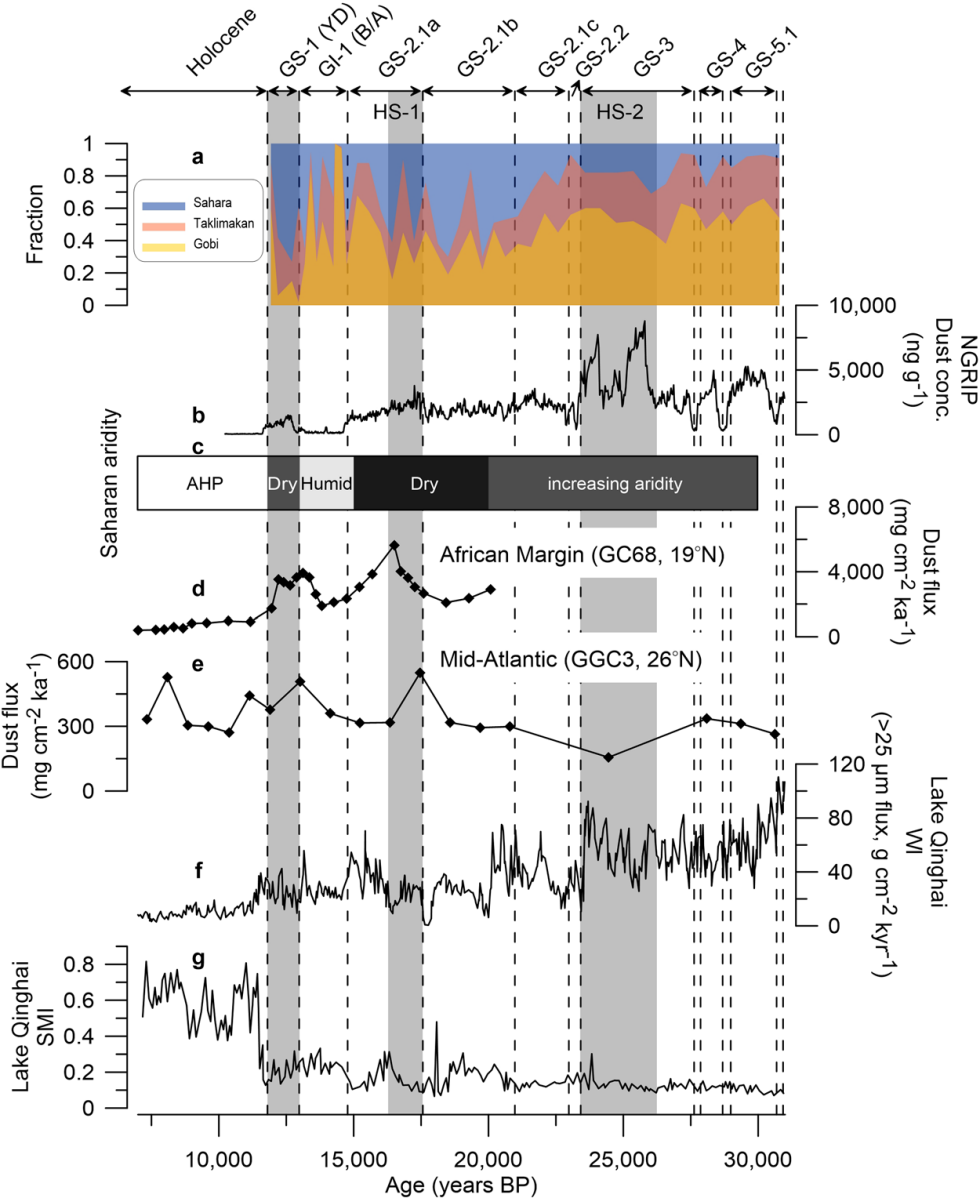
Changes in the relative contributions of the Taklimakan, the Gobi and the Sahara to the Greenland dust deposition from the YD to the GS-5.1 together with paleoclimate proxy records. (**a**) The relative source strength (in fraction) of the three PSAs calculated using a mixing isotopic model (see Methods and Supplementary Table S2). Also shown are Greenland climatic events^25^. (**b**) The NGRIP Greenland ice core dust concentration record^34^. (**c**) Time intervals of Saharan aridity and humidity^39–42^ (see text). AHP indicates the African Humid Period. (**d**,**e**) Dust flux records from the northwest African margin site GC68 (19.36° N, 17.28° W)^42^ and the Mid-Atlantic site GGC3 (26.14° N, 44.80° W)^41^, respectively. (**f**,**g**) Lake Qinghai (36.32°–37.15° N, 99.36°–100.47° E), situated on the northeastern Tibetan Plateau, Westerlies climate index (WI, flux of >25 μm fraction) and Asian summer monsoon index (SMI)^35^, respectively. The non-dimensional Lake Qinghai SMI was quantified by normalizing and averaging two Asian summer monsoon (ASM) proxies (CaCO_3_ and total organic carbon flux) in the lake sediments, representing larger SMI values as the stronger ASM^35^.

We made a rough estimate of the magnitude of dust contribution from the Taklimakan, the Gobi and the Sahara using a mixing isotopic model (see Methods and Fig. 4a). Our modeling approach shows on average a 85% (69–94%) contribution from the Taklimakan and the Gobi prior to GS-2.1, during which dust concentrations were relatively higher (Fig. 4a,b and Supplementary Table S2). This contribution remains still high at an average of 71% (55–83%) during GS-2.1c. Subsequently, the Sahara becomes a more important contributor during GS-2.1b, in line with the significant reduction of dust concentrations, accounting for an average of 49% (16–70%) of Greenland glacial dust (Fig. 4a,b). During GS-2.1a, the Saharan contribution accounts for 36% (11–61%) and shows a continuous decline to 18% (0–62%) in the warmer climatic conditions of the early B/A period. Conversely, significantly enhanced Saharan contributions with an average of 49% (12–73%) are observed from the late B/A to the YD periods when the climate becomes colder (Fig. 4a and Supplementary Table S2).

Our model simulation underscores the importance of the Sahara as a dust contributor to Greenland glacial dust during certain glacial stages, possibly due to intensification of the Saharan aridity coupled with atmospheric circulation changes in the North Atlantic (see the next section). Given the importance of the Saharan contribution over the study period, our results suggest that the dust cycle in northern high latitudes is sensitive to climate and climate-related environmental changes eventually affecting emissions and long-range transport of dust from different sources^12,33^. Finally, our findings potentially provide opportunities for improved resolution of paleoclimate modeling to simulate the dust effects on direct and indirect radiative forcing linked to the abrupt changes observed during glacial epochs.

### Potential climate-induced causes for the enhanced Saharan dust inputs

To infer first-order insights into the potential causes enhancing Saharan dust deposition, we compare our model simulation to paleoclimate proxy records from the tropical North Atlantic sediments and Chinese lake sediments (Fig. 4). However, this approach may have limited applicability, because the proxy-based records have a lower time resolution and may reflect site-specific characteristics.

Dominant dust supply from East Asian sources appears during GS-3 to GS-5.1, characterized by the pronounced increase of Greenland dust concentrations^34^ (Fig. 4a,b). The flux of the >25 μm fraction as a Westerlies climate index (WI) in Lake Qinghai sediments exhibits relatively higher values during the corresponding periods^35^ (Fig. 4f). High WI values indicate strengthened Westerlies influence and intensified aridification over inland East Asia^35^, and thus the WI sequence suggest a remarkable increase of wind intensity and dustiness in East Asia during GS-3 to GS-5.1^35–38^. Conversely, a wetter phase, relative to the present day, occurred between >40 and 25 kyr BP in the Sahara, probably due to more frequent southerly-shifted North Atlantic winter cyclones and/or a northern advance of the African summer monsoon during cold periods^39,40^. Thus, we infer that the stronger westerly winds and source intensification in the East Asian source areas might have caused a dominant East Asian contribution during periods prior to GS-2.1, when Saharan dust sources were relatively inactive.

After HS-2, the Saharan contribution became significant during GS-2.1, particularly GS-2.1b (Fig. 4a). During these periods, the Lake Qinghai WI exhibits lower values (Fig. 4f), albeit with fluctuations, which reflect weakened Westerlies^35^. Comparatively, the Lake Qinghai Summer Monsoon Index (SMI) shows slightly increased values during GS-2.1b (Fig. 4g), and this enhancement represents slight strengthening of the Asian summer monsoon (ASM)^35^. These patterns of the Lake Qinghai WI and SMI are generally consistent with mode-based estimates of the magnitude of Saharan dust contribution changes associated with GS-2.1 (Fig. 4). Accordingly, the weakening of the Westerlies, coupled with the strengthened ASM, may have led to decreased dust storminess in East Asian deserts and a resultant reduction of the East Asian contribution to Greenland dust deposition. The slightly lowered dust concentration level compared to GS-2.1c and HS-1 supports this hypothesis (Fig. 4b). By comparison, the sediment record from the tropical North Atlantic (Fig. 4e), reflecting major fluctuations in Saharan dust deposition^41,42^, displays a steady increase in dust fluxes over the GS-2.1. This substantial change in Saharan dust deposition is coincident with a known increase in the Saharan aridity, reaching its maximum at ~20–15 kyr BP^39–41^ (Fig. 4c).

Finally, the Saharan contribution exhibits a decline during the B/A warm period and subsequent enhancement from the late B/A to the YD cold period (Fig. 4a). This feature matches well with changes in Greenland dust concentrations and Saharan dust deposition in the tropical North Atlantic that are synchronous with the humid phase of the Sahara, initially commenced at ~15 kyr BP, and the following aridity punctuated by the YD^40^, and then the African Humid Period around ~12.3–5.5 kyr BP^40–42^ (Fig. 4b–e). Such synchronous changes strengthen our hypothesis that the intensified aridity in the Sahara is the best candidate as the major influence on enhanced Saharan dust supply to Greenland glacial dust deposition.

Saharan dust inputs to Greenland may have increased by means of the more effective glacial atmospheric dynamics favoring a potential transport through a northward advection. A possible transport route may have passed over the subpolar North Atlantic, moving directly into Greenland, as identified by air mass back trajectory analysis for the Saharan dust events reaching the aerosol sampling campaign sites over central Greenland^43,44^. These events, accompanying a westward motion of Saharan dust plumes by trade winds and subsequent turn northward by the westerly flow, are caused by the propagating low pressure systems over the North Atlantic^45^. During the LGM, the stronger than present southwesterly winds prevailed over the subpolar North Atlantic in association with a southward shift of the generally deeper Icelandic low^46^. Such LGM circulation changes may have important effects on facilitating more frequent Saharan dust transport to Greenland through the subpolar North Atlantic^33^.

To summarize, our results represent the first distinct evidence for a potential Saharan contribution to Greenland dust during the cold stages over the last ~31 kyr. We suggest that changes in the relative source strength between East Asian deserts and the Sahara are likely associated with combined aspects of the climatic conditions in different source areas and transport efficiency closely tied to atmospheric circulation changes during the LLGA. Further studies, extending back to the full glacial period (~100 kyr BP), will provide new insights into climate-related changes in dust provenance, transport mechanisms and vertical dust distribution patterns in northern high latitudes, and the resultant climate feedback processes, particularly linked to the past Arctic amplification over the full glacial age.

## Methods

### Ice core samples

We selected 67 samples from the 2,540 m NEEM ice core drilled at the northwest site, Greenland^24^. The depth of these samples ranged from 1232.0 to 1671.65 m, which corresponds to the age of ~8,260 to ~30,800 years BP, respectively. This time interval is well characterized by a series of abrupt climate changes known as Dansgaard-Oeschger (D/O) events and Heinrich events^10,11,24^. Twenty-two samples were selected for the early Holocene (~8,260 to 11,540 years BP), 3 for the Younger Dryas cooling period (~11,930 to ~12,700 years BP) corresponding to Greenland Stadial-1 (GS-1), 8 for the Bølling-Allerød (~12,950 to 14,550 years BP) warming period, Greenland Interstadial-1 (GI-1), and 34 for GS-2 to GS-5.1 (~14,740 to ~30,790 years BP), including the LGM (~19,000 to 23,000 years BP) of the LLGA. In our study, the climatic event stratigraphy was defined by the sequence of GS and GI, reflecting the characteristic sequence of Greenland climate changes based on robust correlation between different proxy climate records from the NGRIP, GRIP, and GISP2 Greenland ice cores^25^. Each core section (20 cm in length) with a cross section of 4 × 4 cm^2^ was mechanically decontaminated using an ultraclean decontamination method that we have developed for isotopic analysis of small volumes of the NEEM ice (see details in ref.^26^). It involves the chiseling of successive veneers of ice from the outside of the core toward the center to obtain the uncontaminated inner part of the core section, using ultraclean stainless steel chisels or ceramic knives, inside a Class 100 vertical laminar flow clean bench installed inside a cold room at the Korea Polar Research Institute (KOPRI), Korea. This ultraclean decontamination method allowed the determination of Pb and Sr isotopes even in the NEEM ice core sections, which contain only a few tens of picograms of Pb and Sr in a sample weight of ~10 g^26^. The inner core samples were divided into two pieces, each 10 cm long (a sample volume of ~80 mL), and the upper piece was analyzed for this study. Each subsample provides a span time ranging from ~0.5 year (1232.0 m) to ~2 years (1671.65 m) of snow accumulation. Thus, it enabled us to obtain sufficiently high-resolution time records of Pb and Sr isotopes compared to previous methods for isotopic measurements of Nd, Sr and Pb in a few Greenland glacial samples with sample weights ranging from ~0.5 to 8 kg^16–19^.

### Isotopic measurements by thermal ionization mass spectrometry

All sample handling and ultraclean analytical procedures for determining elemental concentrations and isotopic analysis were performed under a Class 10 vertical laminar flow clean bench or booth in clean laboratories (Class 1000) at KOPRI, as described in detail in ref.^26^. The Pb and Ba concentrations and Pb isotopic composition were simultaneously analyzed using a thermal ionization mass spectrometry (TIMS; TRITON, Thermo Scientific) fitted with a 23 cm radius, 90° magnetic sector containing a 21-sample carousel. The samples (~10 g) were first evaporated to dryness with a mixture of 10 μL of 65% HNO_3_ (Fisher “Optima” ultrapure grade), 20 μL of 48% HF (Merck “Ultrapur” grade), and 4 μL of dilute H_3_PO_4_ (Merck “Suprapur” grade; ~5% by weight), together with 10 μL of a mixed tracer solution containing accurately known amounts of the enriched isotopes ^204^Pb and ^137^Ba, on a Teflon-coated hot plate at ~80 °C. A droplet of PL-7 silica-gel activator was then dropped into the evaporated residue and a mixture of the sample residue and silica-gel was transferred to a degassed (4 A, 30 min), zone-refined rhenium filament (99.999% Re, 0.7 mm wide, 0.04 mm thick, H. Cross Company). The addition of the ^204^Pb and ^137^Ba spikes to the sample enabled both the Pb and Ba concentrations, using isotope dilution mass spectrometry, and the Pb isotopic composition to be determined in a single measurement. The final isotopic ratios were obtained after correction for the blanks. The accuracy for the concentrations was better than 10% (95% confidence interval) and the precision was ~0.28% for Pb isotope ratios on a small sample size (tens of picograms of Pb)^26^.

For Sr isotopes, dust particles in the sample (10 mL) were separated by centrifugation at 4,000 RPM for 10 min and the top solution of 6 mL was then removed by using a pipette and 6 mL of ultrapure Millipore Milli-Q water (MQW) was added. These two steps were repeated 5 times. Because carbonate minerals (mainly calcite) have higher Sr contents and significantly less radiogenic Sr isotopic ratios relative to aluminosilicates^17^, samples of the dust fraction were dissolved for 8 hours at room temperature using 2 mL of 0.5 M buffered ultrapure acetic acid to remove insoluble carbonate minerals in the dust fraction. The resulting aluminosilicate fraction was then separated by centrifugation with multiple washings in MQW. The aluminosilicates were dissolved in a HNO_3_/HF mixture and then evaporated to dryness on a hotplate at ~80 °C. The residue was re-dissolved in ~500 μL of 3.5 M HNO_3_ and Sr was isolated on an ion-exchange column with Sr-Spec resin (Eichrom Industries, IL, USA)^17^. The samples were added to the column and washed with 3.5 N HNO_3_. Sr was then eluted with sub-boiling distilled ultrapure water by a sub-boiling distillation system with two high-purity quartz distillation units (Milestone, DuoPUR) using the MQW, which renders more than 90% of Sr recovered with negligible Rb interference^17^. Finally, 2 μL of 4% H_3_PO_4_ was added to the Sr eluants before evaporation to easily identify the sample in the beaker. They were loaded onto degassed Re filaments with Ta_2_O_5_ activator solution. The Sr isotopic composition was determined using a TRITON TIMS for both the insoluble dust (no pretreatment by buffered acetic acid) and the carbonate-free dust (aluminosilicates) in the same samples. Our analytical procedures ensured a precision of ~0.05% for the ^87^Sr/^86^Sr ratios at the lowest Sr concentration.

The Sr concentrations in the sample were directly determined by inductively coupled plasma sector field mass spectrometer (ICP-SFMS; Element2, Thermo Scientific), coupled with an APEX micro-nebulization desolvation system (APEX IR, HF, ESA, USA).

### Mixing isotopic model for calculating the relative source strength

The relative contribution of each dust source to the dust deposition on Greenland was estimated using a mixing isotopic model between dusts exported from the Sahara, the Taklimakan and the Gobi deserts, assuming that they are the three major PSAs dominating Greenland glacial dust. The isotopic contribution of each PSA in the samples can be estimated by the following mixing equations:

1 $${(\frac{{}^{206}Pb}{{}^{207}Pb})}_{m}={(\frac{{}^{206}Pb}{{}^{207}Pb})}_{S}\times {F}_{Pb,S}+{(\frac{{}^{206}Pb}{{}^{207}Pb})}_{T}\times {F}_{Pb,T}+{(\frac{{}^{206}Pb}{{}^{207}Pb})}_{G}\times {F}_{Pb,G}$$

2 $${(\frac{{}^{208}Pb}{{}^{207}Pb})}_{m}={(\frac{{}^{208}Pb}{{}^{207}Pb})}_{S}\times {F}_{Pb,S}+{(\frac{{}^{208}Pb}{{}^{207}Pb})}_{T}\times {F}_{Pb,T}+{(\frac{{}^{208}Pb}{{}^{207}Pb})}_{G}\times {F}_{Pb,G}$$

3 $${(\frac{{}^{87}Sr}{{}^{86}Sr})}_{m}={(\frac{{}^{87}Sr}{{}^{86}Sr})}_{S}\times {F}_{Sr,S}+{(\frac{{}^{87}Sr}{{}^{86}Sr})}_{T}\times {F}_{Sr,T}+{(\frac{{}^{87}Sr}{{}^{86}Sr})}_{G}\times {F}_{Sr,G}$$

where *m* indicates the measured isotopic value, *S*, *T* and *G* are the isotopic ratio of end-member of the Sahara, the Taklimakan and the Gobi, respectively, and *F* represents the fractional contribution of each end-member for Pb (*F*_*Pb*_) and Sr (*F*_*Sr*_) with *F*_*S*_ + *F*_*T*_ + *F*_*G*_ = 1. Proportions of the relative contribution of the individual PSAs were addressed for the 45 samples from the YD to GS-5.1 by means of a Monte Carlo inversion approach consisting of two steps. First, the isotopic composition of each end-member was randomly selected within the ranges depicted in Figs 2 and 3. Then, the Genetic Algorithm embedded in the Matlab Optimization Toolbox is used to seek the best *F* values resulting in minimum errors in the above equations. Uncertainties of 0.01, 0.02, and 0.001 for ^206^Pb/^207^Pb, ^208^Pb/^207^Pb and ^87^Sr/^86^Sr, respectively, were allowed in the measured isotopic values and weighted in the total error calculation. These two steps were repeated more than 100,000 times. To render the simultaneous equations solvable, the Pb/Sr ratios of each end-member are assumed to be identical (*F*_*Pb*_ = *F*_*Sr*_). The resulting model approach yields a temporal record of the relative importance of the Sahara, the Taklimakan and the Gobi deserts as sources to the Greenland glacial dust. Considering a wider range of end-member values away from the sample values for the Taklimakan Sr-Pb isotopic compositions relative to those for the Gobi, our model simulation would potentially overestimate the magnitude of dust contribution from the Gobi.

## Electronic supplementary material

Supplementary materials

Dataset 1
