# Supplementary material for: High-resolution isotopic evidence for a potential Saharan provenance of Greenland glacial dust
Source: Sci Rep. 2018 Oct 22;8:15582. doi: 10.1038/s41598-018-33859-0 (PMC6197225; doi:10.1038/s41598-018-33859-0)
Supplement: Supplementary file 1 — Supplementary materials [file 41598_2018_33859_MOESM1_ESM.docx]

**Supplementary materials**

**High-resolution isotopic evidence for a potential Saharan provenance of Greenland glacial dust**

Changhee Han^1,2^, Soon Do Hur^2^, Yeongcheol Han^2^, Khanghyun Lee^2^, Sungmin Hong^1,*^ , Tobias Erhardt^3^, Hubertus Fischer^3^, Anders M. Svensson ^4^, Jørgen Peder Steffensen^4^, Paul Vallelonga^4^

^1^Department of Ocean Sciences, Inha University, 100 Inha-ro, Nam-gu, Incheon 22212, Korea

^2^Korea Polar Research Institute, 26 Songdomirearo, Yeonsu-gu, Incheon 21990, Korea

^3^Climate and Environmental Physics, Physics Institute & Oeschger Center for Climate Change Research, University of Bern, Sidlerstrasse 5, 3012 Bern, Switzerland

^4^Center for Ice and Climate, Niels Bohr Institute, University of Copenhagen, Julian Maries Vej 30, 2100 Copenhagen, Denmark

Corresponding author: *Email:* smhong@inha.ac.kr (Sungmin Hong); *Phone*: +82-32-860-7708; *Fax*: +82-32-862-5236

Table S1. Elemental concentrations and Pb and Sr isotopic compositions measured in NEEM ice core (Table S1 is provided as a separate Excel file).

Table S2. The estimated magnitude of dust contribution from the Taklimakan, the Gobi, and the Sahara during different climatic periods

| Period | Age (years BP) |  | Taklimakan | Gobi | Sahara |
| --- | --- | --- | --- | --- | --- |
|  |  |  |  |  |  |
| GI-1  (Late B/A)  to  GS-1 (YD) | 11,932–13,186 | Mean ± SD | 0.29 ± 0.20 | 0.22 ± 0.23 | 0.49 ± 0.24 |
|  |  | Min–max | 0.09–0.59 | 0.02–0.60 | 0.12–0.73 |
| GI-1  (early B/A) | 13,411–14,551 | Mean ± SD | 0.18 ± 0.19 | 0.64 ± 0.35 | 0.18 ± 0.24 |
|  |  | Min–max | 0.00–0.43 | 0.24–1.00 | 0.00–0.62 |
| GS-2.1a | 14,737–17,240 | Mean ± SD | 0.24 ± 0.11 | 0.40 ± 0.19 | 0.36 ± 0.24 |
|  |  | Min–max | 0.14–0.45 | 0.16–0.68 | 0.11–0.61 |
| GS-2.1b | 17,666–20,621 | Mean ± SD | 0.17 ± 0.12 | 0.34 ± 0.11 | 0.49 ± 0.20 |
|  |  | Min–max | 0.04–0.37 | 0.19–0.47 | 0.16–0.70 |
| GS-2.1c | 21,087–22,578 | Mean ± SD | 0.27 ± 0.07 | 0.44 ± 0.09 | 0.29 ± 0.12 |
|  |  | Min–max | 0.17–0.34 | 0.36–0.57 | 0.17–0.45 |
| GS-2.2 to GS-5.1 | 23,046–30,787 | Mean ± SD | 0.30 ± 0.05 | 0.55 ± 0.07 | 0.15 ± 0.08 |
|  |  | Min–max | 0.22–0.37 | 0.38–0.66 | 0.06–0.31 |

Figure S1. Pb isotopic compositions in PSA samples from published literature: Alaskan loess in Fairbanks and US loess in Illinois and Washington (<5 µm)^1^, the Sahara desert from the tropical to subtropical Atlantic sediments (bulk)^2^, Bodélé Depression (the southern edge of the Sahara desert, bulk)^3^, Chinese loess (<5 µm)^1^, the Taklimakan desert (<5 µm)^4-6^, the Gobi desert (<5 µm)^1^, Northern China and Ordos Plateau (bulk)^4^, North Atlantic sediment data (SE and SW of Greenland, <63 µm)^7^, Nussloch loess in Germany (bulk)^8^, Eastern German loess (bulk)^8^, Eastern European loess (bulk)^8^, Western European loess (bulk)^8^, Spitsbergen volcanic data^9,10^, Laacher See volcanic data in Germany^11^, Mexican volcanic data in central Mexico^12^, Japanese volcanic data^13-16^, Aleutian island arc^17^, Pacific and Atlantic mid-ocean ridge basalt (MORB)^18,19^, Italian volcanic data^20^, Kamchatka arc^21^, Iceland volcanic data^22^.

**
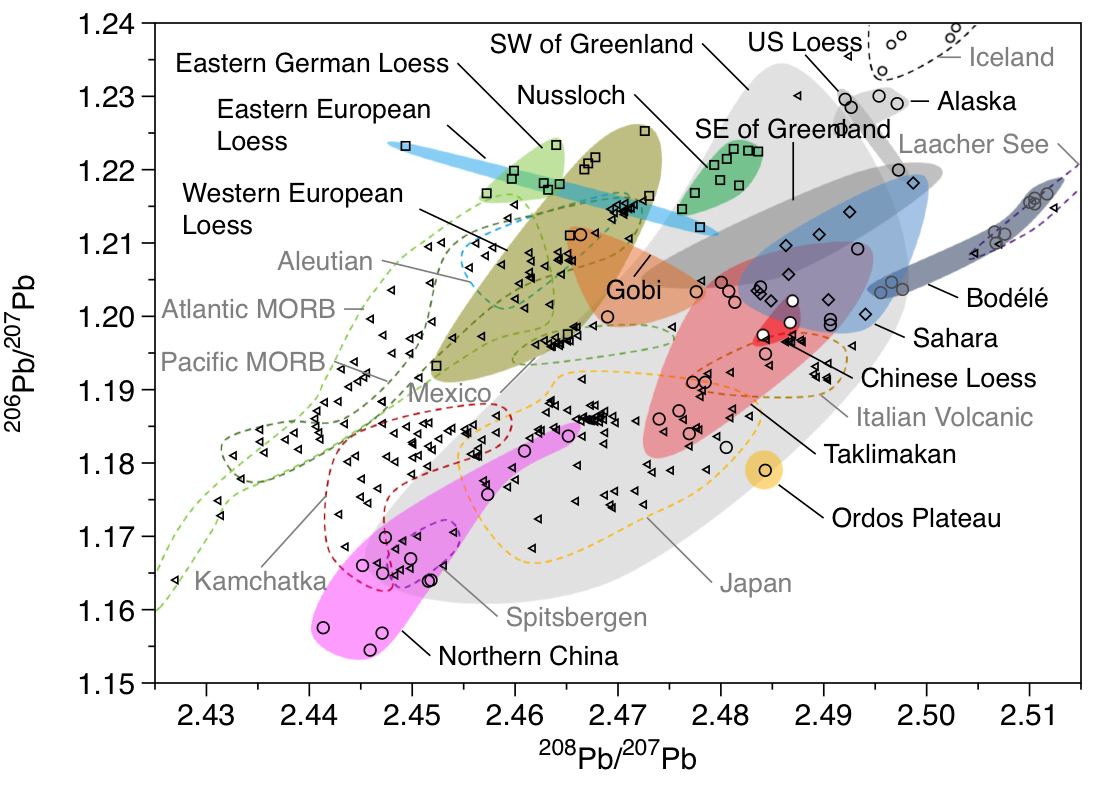
**

Figure S2. Sr and Pb isotopic compositions in PSA samples from published literature: Alaskan loess in Fairbanks and US loess in Illinois and Washington (<5 µm)^1^, the Sahara desert from the tropical to subtropical Atlantic sediments (bulk^2^, <30 µm^23^) and aerosol dust (<30 µm^23,24^, <1 µm^25^), Bodélé Depression (the southern edge of the Sahara desert, bulk)^3^, Chinese loess (<5 µm)^1^, the Taklimakan desert (<5 µm)^6,26,27,28^, the Gobi desert (<5 µm)^1^, Northern China and Ordos Plateau (<5 µm)^4,26^, North Atlantic sediment data (<63 µm)^7^, Nussloch loess in Germany (bulk)^8^, Eastern German loess (bulk)^8^, Eastern European loess (bulk)^8^, Western European loess (bulk)^8^, Spitsbergen volcanic data^9,10^, Mexican volcanic data in central Mexico^12^, Japanese volcanic data^13-16^, Aleutian island arc^17^, Pacific and Atlantic mid-ocean ridge basalt (MORB)^18,19^, Italian volcanic data^20^, Kamchatka arc^21^, Iceland volcanic data^22^.

**
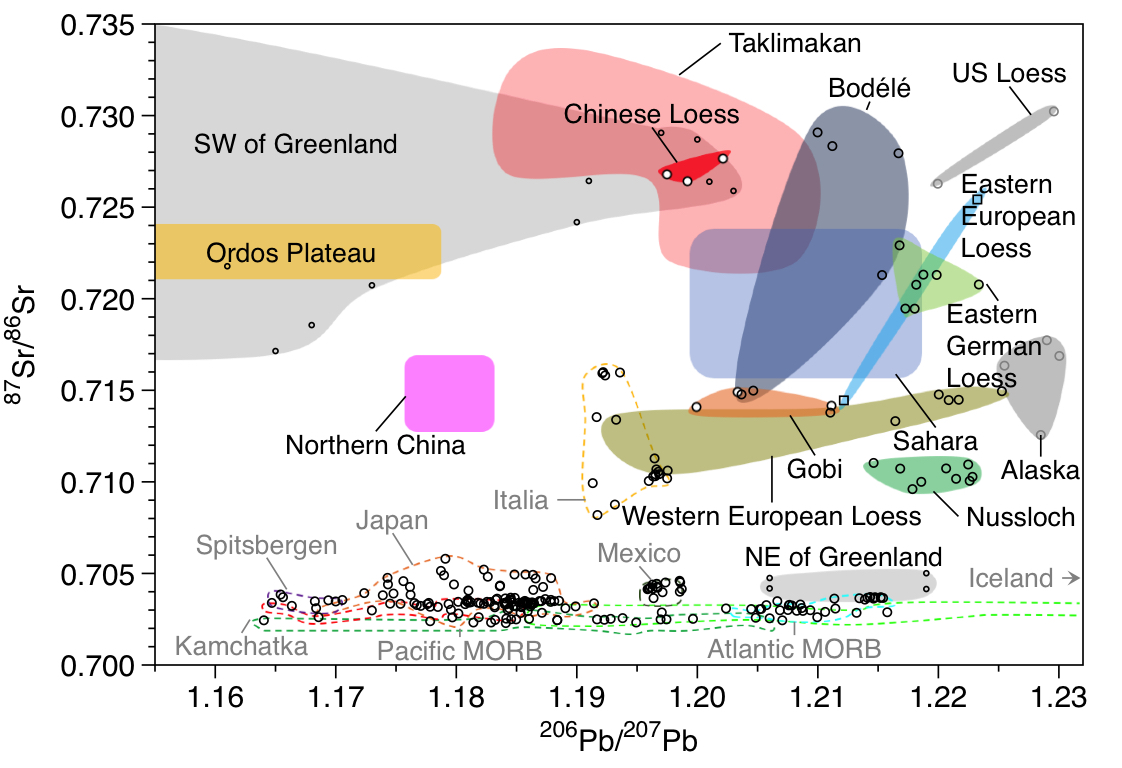
**

Figure S3. Pb and Sr isotopic compositions measured in NEEM ice core samples. Samples for the early Holocene, the Younger Dryas and the Bølling-Allerød are in (a) and (b), samples for GS-2.1a to GS-2.1c in (c) and (d), samples for GS-2.2 to GS-5 in (e) and (f). Sample numbers are shown in the first column of Table S1.


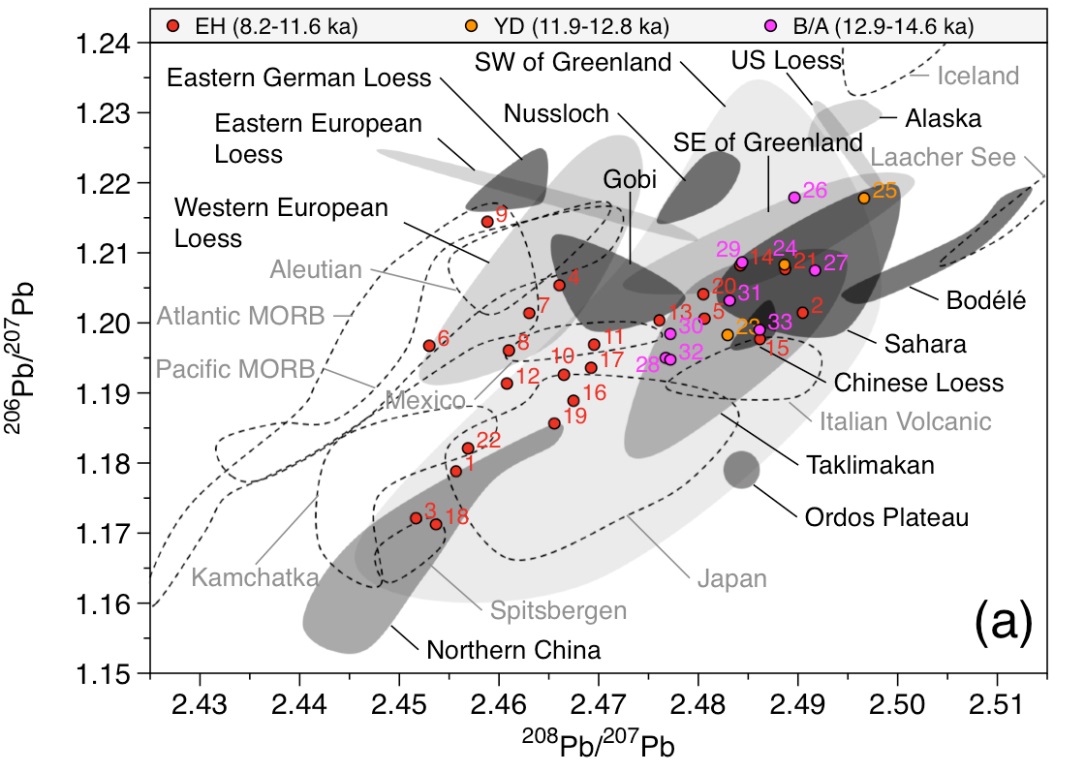


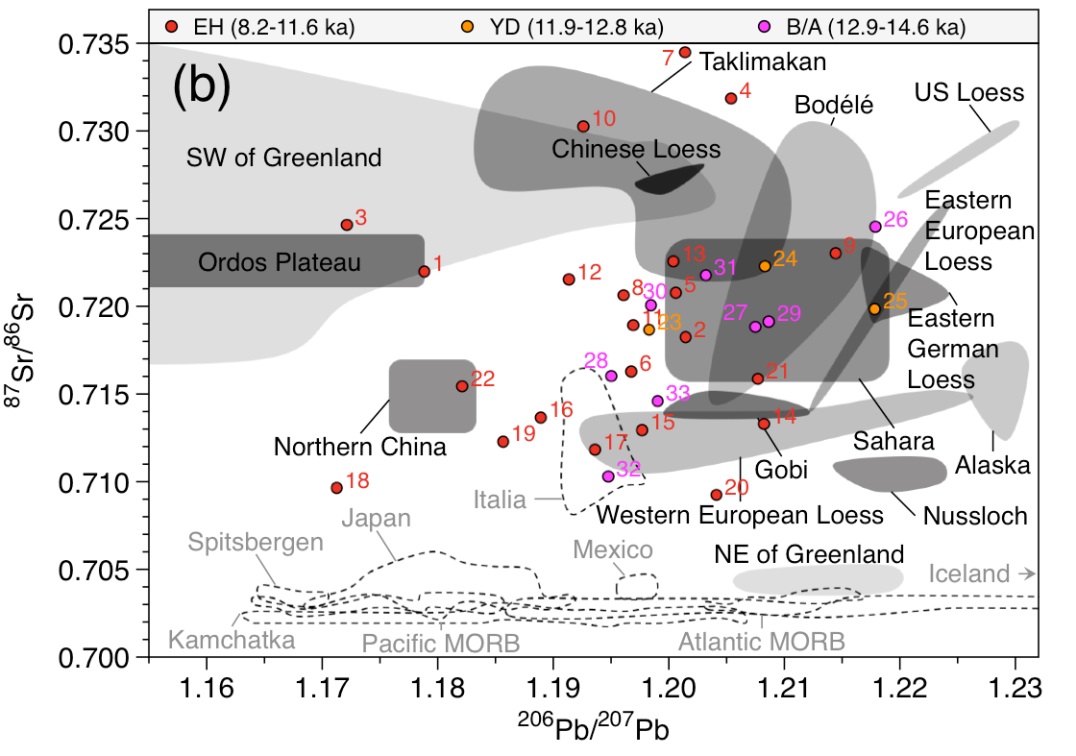


Figure S3. (continued).


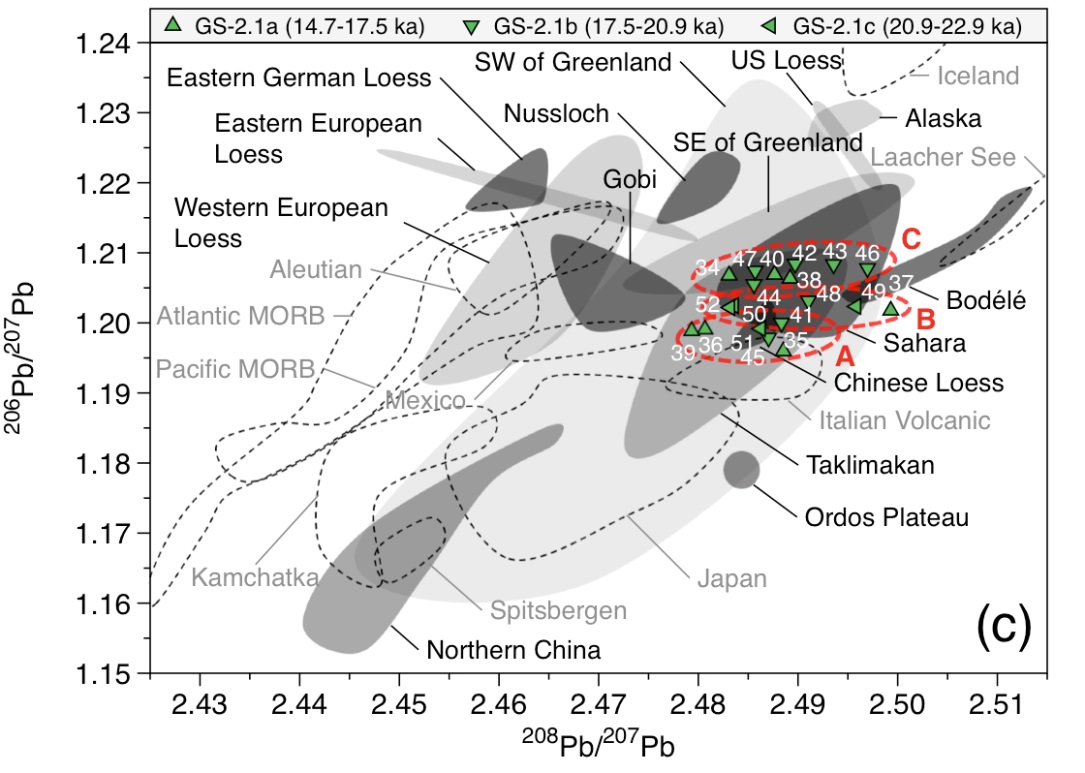


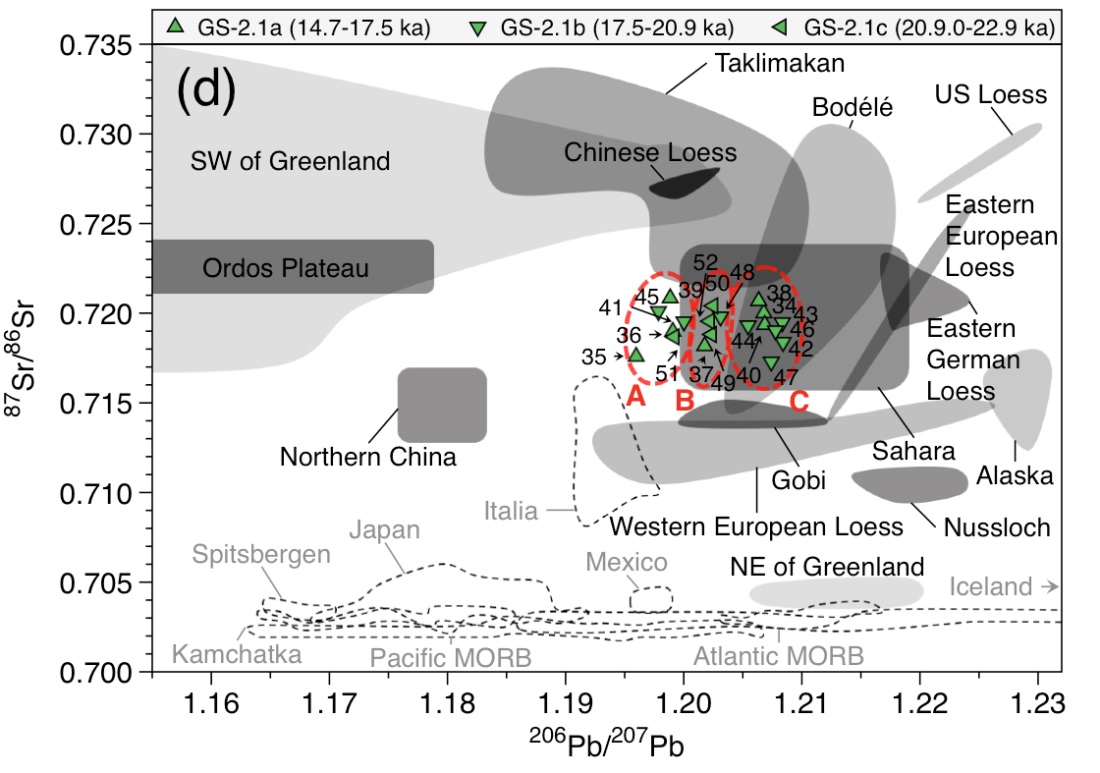


Figure S3. (continued).


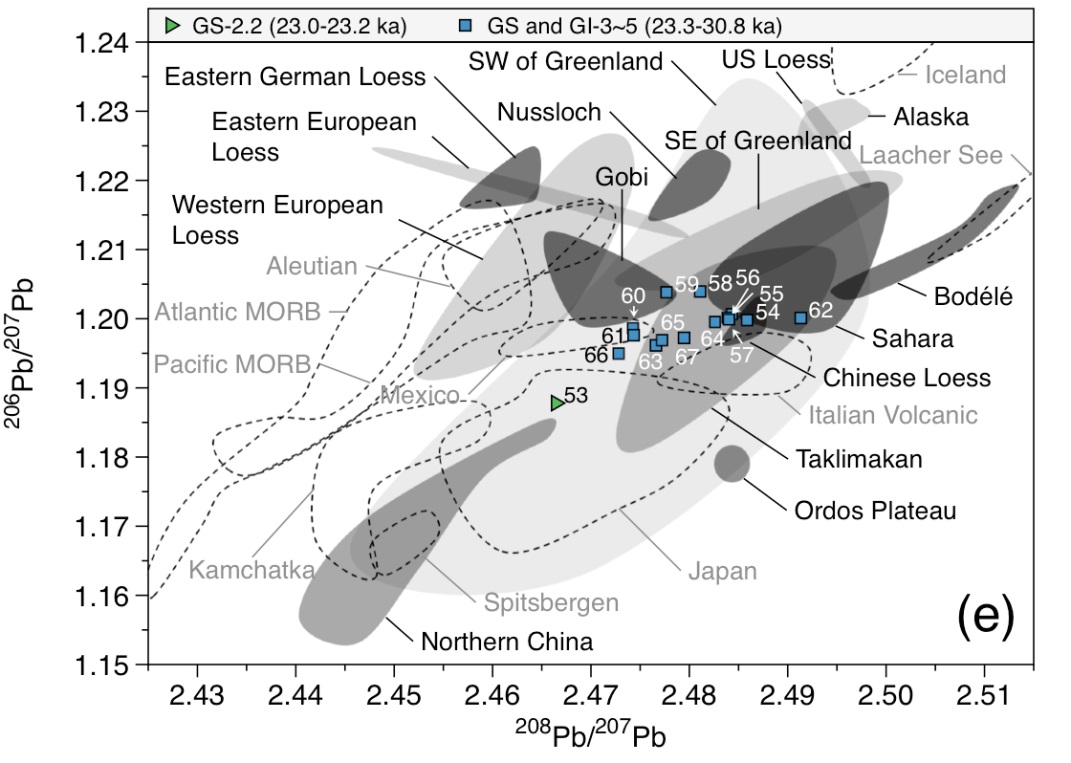


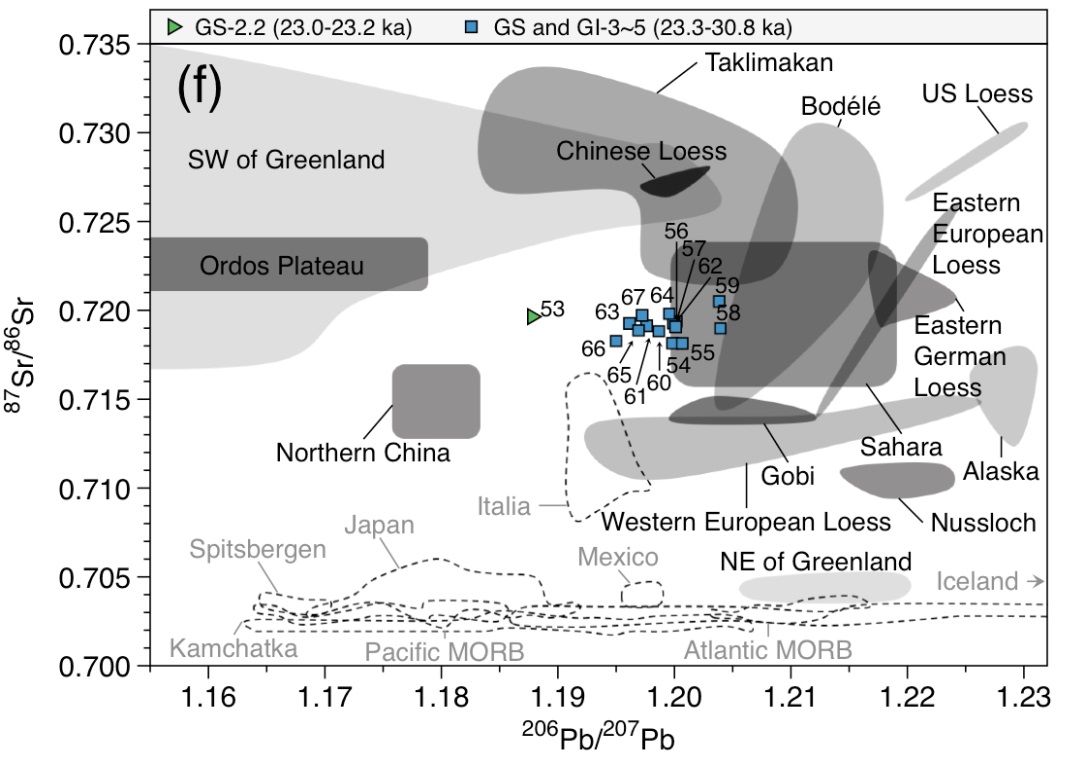


Figure S4. Comparison of ^87^Sr/^86^Sr ratios between insoluble (open circles) and aluminosilicate (solid circles) fractions with Sr concentrations (open triangles). Insoluble fraction was separated by removing melted ice and aluminosilicate fraction was pretreated by buffered acetic acid (see Methods). The Sr isotopic composition in aluminosilicates is more radiogenic than those in insoluble fraction, because carbonate minerals have less radiogenic Sr isotopic ratios relative to aluminosilicates.


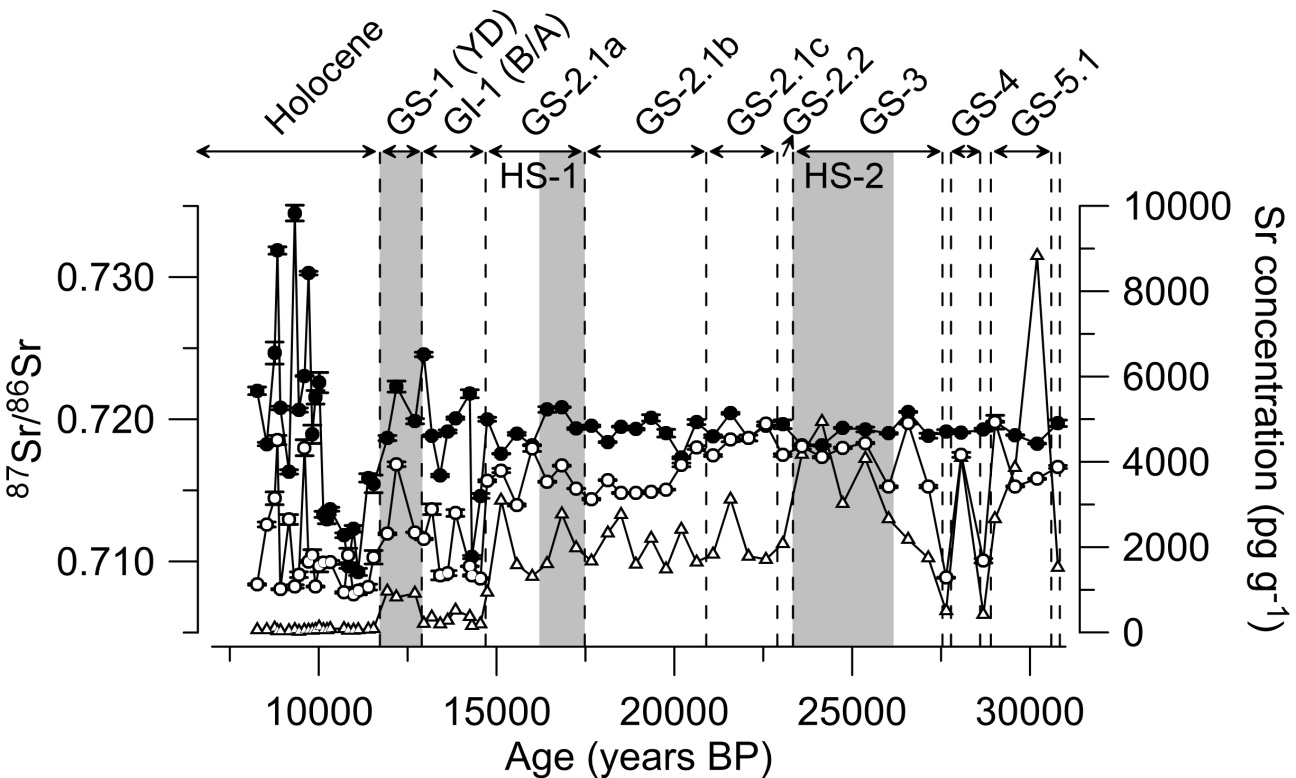


**References**

1. Biscaye, P. E. *et al*. Asian provenance of glacial dust (stage 2) in the Greenland Ice Sheet Project 2 Ice Core, Summit, Greenland. *J. Geophys. Res.* **102,** 26765–26781 (1997).
2. Sun, S. –S. Lead isotopic study of young volcanic rocks from mid-ocean ridges, ocean islands and island arcs. *Phil. Trans. R. Soc. A.* **297,** 409–445 (1980).
3. Abouchami, W. *et al.* Geochemical and isotopic characterization of the Bodélé Depression dust source and implications for transatlantic dust transport to the Amazon Basin. *Earth Planet. Sci. Lett.* **380,** 112–123 (2013).
4. Li, F. Distribution characteristics of lead isotope in dust source areas and its trace significance in the North of China. *J. Desert Res.* **27**, 738–744 (2007), in Chinese.
5. Ferrat, M. *et al*. Lead atmospheric deposition rates and isotopic trends in Asian dust during the last 9.5kyr recorded in an ombrotrophic peat bog on the eastern Qinghai-Tibetan Plateau. *Geochim. Cosmochim. Acta* **82,** 4–22 (2012).
6. Bory, A. J. M. *et al*. A Chinese imprint in insoluble pollutants recently deposited in central Greenland as indicated by lead isotopes. *Environ. Sci. Technol.* **48,** 1451–1457 (2014).
7. Farmer, G. L., Barber, D. & Andrews, J. Provenance of Late Quaternary ice-proximal sediments in the North Atlantic: Nd, Sr and Pb isotopic evidence. *Earth Planet. Sci. Lett.* **209,** 227–243 (2003).
8. Rousseau, D. D. *et al*. European glacial dust deposits: Geochemical constraints on atmospheric dust cycle modeling. *Geophys. Res. Lett.* **41**, 7666-7674 (2014).
9. Sushchevskaya, N. M., Evdokimov, A. N., Belyatsky, B. V., Maslov, V. A. & Kuz’min, D. V. Conditions of quaternary magmatism at Spitsbergen Island. *Geochem. Int.* **46**, 1–16 (2008).
10. Ionov, D. A., Mukasa, S. B. & Bodinier, J. –L. Sr–Nd–Pb isotopic compositions of peridotite xenoliths from Spitsbergen: Numerical modelling indicates Sr–Nd decoupling in the mantle by melt percolation metasomatism. *J. Petrol.* **43,** 2261–2278 (2002).
11. Wörner, G., Zindler, A., Staudigel, H. & Schmincke, H. –U. Sr, Nd, and Pb isotope geochemistry of Tertiary and Quaternary alkaline volcanics from West Germany. *Earth Planet. Sci. Lett.* **79**, 107–119 (1986).
12. Schaaf, P., Stimac, J., Siebe, C. & Macías, J. L. Geochemical evidence for mantle origin and crustal processes in volcanic rocks from Popocatépetl and surrounding monogenetic volcanoes, central Mexico. *J. Petrol.* **46,** 1243–1282 (2005).
13. Hedge, C. E. & Knight, R. J. Lead and strontium isotopes in volcanic rocks from northern Honshu, Japan. *Geochem. J.* **3,** 15–24 (1969).
14. Shinjo, R., Woodhead, J. D. & Hergt, J. M. Geochemical variation within the northern Ryukyu Arc: magma source compositions and geodynamic implications. *Contrib. Mineral. Petrol.* **140,** 263–282 (2000).
15. Hoang, N., Itoh, J. & Miyagi, I. Subduction components in Pleistocene to recent Kurile arc magmas in NE Hokkaido, Japan. *J. Volcanol. Geotherm. Res.* **200,** 255–266 (2011).
16. Taylor, R. N. & Nesbitt, R. W. Isotopic characteristics of subduction fluids in an intra-oceanic setting, Izu–Bonin Arc, Japan. *Earth Planet. Sci. Lett.* **164,** 79–98 (1998).
17. Jicha, B. R. *et al*. Variable impact of the subducted slab on Aleutian island arc magma sources: evidence from Sr, Nd, Pb, and Hf isotopes and trace element abundances. *J. Petrol.* **45**, 1945–1875 (2004).
18. Ito, E., White, W. M. & Göpel, C. The O, Sr, Nd and Pb isotope geochemistry of MORB. *Chem. Geol.* **62**, 157–176 (1987).
19. White, W. M., Hofmann, A. W. & Puchelt, H. Isotope geochemistry of Pacific mid‐ocean ridge basalt. *J. Geophys. Res.* **92**, 4881–4893 (1987).
20. Conticelli, S., D'Antonio, M., Pinarelli, L. & Civetta, L. Source contamination and mantle heterogeneity in the genesis of Italian potassic and ultrapotassic volcanic rocks: Sr–Nd–Pb isotope data from Roman Province and Southern Tuscany. *Mineral. Petrol.* **74,** 189–222 (2002).
21. Kepezhinskas, P. *et al*. Trace element and Sr-Nd-Pb isotopic constraints on a three-component model of Kamchatka Arc petrogenesis. *Geochim. Cosmochim. Acta* **61**, 577–600 (1997).
22. Stecher, O., Carlson, R. W. & Gunnarsson, B. Torfajökull: a radiogenic end-member of the Iceland Pb-isotopic array. *Earth Planet. Sci. Lett*. **165**, 117-127 (1999).
23. Grousset, F. E. *et al*. Saharan wind regimes traced by the Sr–Nd isotopic composition of subtropical Atlantic sediments: last glacial maximum vs today. *Quat. Sci. Rev.* **17,** 395–409 (1998).
24. Grousset, F. E. & Biscaye, P. E. Tracing dust sources and transport patterns using Sr, Nd and Pb isotopes. *Chem. Geol.* **222**, 149-167 (2005).
25. Aarons, S. M., Aciego, S. M. & Gleason, J. D. Variable Hf–Sr–Nd radiogenic isotopic compositions in a Saharan dust storm over the Atlantic: implications for dust flux to oceans, ice sheets and the terrestrial biosphere. *Chem. Geol.* **349-350**, 18-26 (2013).
26. Chen, J. *et al*. Nd and Sr isotopic characteristics of Chinese deserts: implications for the provenances of Asian dust. *Geochim. Cosmochim. Acta* **71**(15), 3904–3914 (2007).
27. Bory, A. J. –M., Biscaye, P. E., Svensson, A. & Grousset, F. E. Seasonal variability in the origin of recent atmospheric mineral dust at NorthGRIP, Greenland. *Earth Planet. Sci. Lett.* **196,** 123–134 (2002).
28. Bory, A. J. –M., Biscaye, P. E. & Grousset, F. E. Two distinct seasonal Asian source regions for mineral dust deposited in Greenland (NorthGRIP). *Geophys. Res. Lett.* **30,** 1167–4 (2003).
